# High versus low attenuation thresholds to determine the solid component of ground-glass opacity nodules

**DOI:** 10.1371/journal.pone.0205490

**Published:** 2018-10-18

**Authors:** Jae Ho Lee, Tae Hoon Kim, Sungsoo Lee, Kyunghwa Han, Min Kwang Byun, Yoon Soo Chang, Hyung Jung Kim, Geun Dong Lee, Chul Hwan Park

**Affiliations:** 1 Department of Radiology and the Research Institute of Radiological Science, Gangnam Severance Hospital, Yonsei University college of Medicine, Seoul, Republic of Korea; 2 Department of Thoracic and Cardiovascular Surgery, Gangnam Severance Hospital, Yonsei University college of Medicine, Seoul, Republic of Korea; 3 Department of Radiology, Research Institute of Radiological Science, Severance Hospital, Yonsei University College of Medicine, Seoul, Republic of Korea; 4 Division of Pulmonology, Department of Internal Medicine, Gangnam Severance Hospital, Yonsei University College of Medicine, Seoul, Republic of Korea; University of South Alabama Mitchell Cancer Institute, UNITED STATES

## Abstract

**Objectives:**

To evaluate and compare the diagnostic accuracy of high versus low attenuation thresholds for determining the solid component of ground-glass opacity nodules (GGNs) for the differential diagnosis of adenocarcinoma in situ (AIS) from minimally invasive adenocarcinoma (MIA) and invasive adenocarcinoma (IA).

**Methods:**

Eighty-six pathologically confirmed GGNs < 3 cm observed in 86 patients (27 male, 59 female; mean age, 59.3 ± 11.0 years) between January 2013 and December 2015 were retrospectively included. The solid component of each GGN was defined using two different attenuation thresholds: high (-160 Hounsfield units [HU]) and low (-400 HU). According to the presence or absence of solid portions, each GGN was categorized as a pure GGN or part-solid GGN. Solid components were regarded as indicators of invasive foci, suggesting MIA or IA.

**Results:**

Among the 86 GGNs, there were 57 cases of IA, 19 of MIA, and 10 of AIS. Using the high attenuation threshold, 44 were categorized as pure GGNs and 42 as part-solid GGNs. Using the low attenuation threshold, 13 were categorized as pure GGNs and 73 as part-solid GGNs. The sensitivity, specificity, positive predictive value, negative predictive value, and diagnostic accuracy for the invasive focus were 55.2%, 100%, 100%, 22.7%, and 60.4%, respectively, for the high attenuation threshold, and 93.4%, 80%, 97.2%, 61.5%, and 91.8%, respectively, for the low attenuation threshold.

**Conclusion:**

The low attenuation threshold was better than the conventional high attenuation threshold for determining the solid components of GGNs, which indicate invasive foci.

## Introduction

According to the new International Association for the Study of Lung Cancer/American Thoracic Society/European Respiratory Society lung adenocarcinoma classifications, adenocarcinoma is pathologically classified as a pre-invasive lesion (atypical adenomatous hyperplasia, adenocarcinoma in situ [AIS]), minimally invasive adenocarcinoma (MIA), or invasive adenocarcinoma (IA) according to the presence and size of the invasive foci [1]. Currently, adenocarcinoma is the most common histological type of lung cancer and often appears as ground-glass opacity nodules (GGNs) on computed tomography (CT) imaging [2].

GGNs are defined on CT as circumscribed lesions with hazy increased attenuation of the lung, but with preservation of the bronchial and vascular margins [3]. GGNs are subcategorized into pure and part-solid types, with part-solid nodules more likely to be primary lung cancer and associated with malignancy rates as high as 63% [4]. For the analysis of GGNs, the presence or absence and the size of the solid portion are important for differential diagnosis, determination of treatment strategy, and risk stratification [5–7]. Although the visual assessment method has been used to categorize GGNs in a few studies, the results have yielded only moderate inter-observer agreement [8–11].

Recently, a few studies have evaluated the utility of objective CT thresholds to determine the solid portion of GGNs [12–14]. These studies have used thresholds varying from -160 Hounsfield units (HU) to -350 HU; however, the optimal threshold for the solid portion of GGNs, which correspond to invasive foci in pathology, has not been established. Therefore, the aims of the present study were to evaluate and compare the diagnostic accuracy of high and low attenuation thresholds for the solid portion of GGN.

## Methods

The present study received approval from the institutional review board of Gangnam Severance Hospital (3-2016-0324). Clinical data were reviewed from medical records. Given the retrospective nature of the study and the use of anonymized data, requirements for informed consent were waived.

### Patients

Patients who underwent thoracic surgery between January 2013 and December 2015 due to persistent GGNs were enrolled and retrospectively reviewed. GGNs were defined as hazy areas of increased attenuation in the lung parenchyma with preserved bronchial and vascular markings on CT imaging. Patients who were diagnosed with AIS, MIA, or IA < 3 cm were included. Patients were excluded if the slice thickness of their pre-operative CT imaging was > 2.5 mm.

### CT protocol

CT scans were performed using one of two multi-detector scanners: a 16-slice (Somatom Sensation 16; Siemens Medical Solutions, Erlangen, Germany) or a 64-slice (Somatom Sensation 64; Siemens Medical Solutions, Erlangen, Germany) device. Scanning was performed during inspiration in patients assuming a supine position. The scanning range was from apex of lung to adrenal gland. After obtaining a scout image to determine the field of view, conventional CT scanning was performed in the mediastinal window setting using a 1–2.5-mm reconstruction interval. The scanning parameters were as follows: voltage, 120 kVp; current, 100–200 mA; slice thickness 1–2.5 mm. CT images were reconstructed using the scanner's workstation. All CT images were retrieved from a picture archiving and communication system (Centricity 2.0, GE Medical Systems, Mt Prospect, IL, USA).

### CT image analysis

Two radiologists (C.H.P. and T.H.K), each with > 10 years of experience with chest radiology interpretation, assessed the CT images. All pre-operative CT images of the enrolled patients were transferred to a commercially available reconstruction program (Aquarius iNtuition Ver. 4.4.6 TeraRecon, Foster City, CA, USA), and then analyzed.

**Fig 1:**
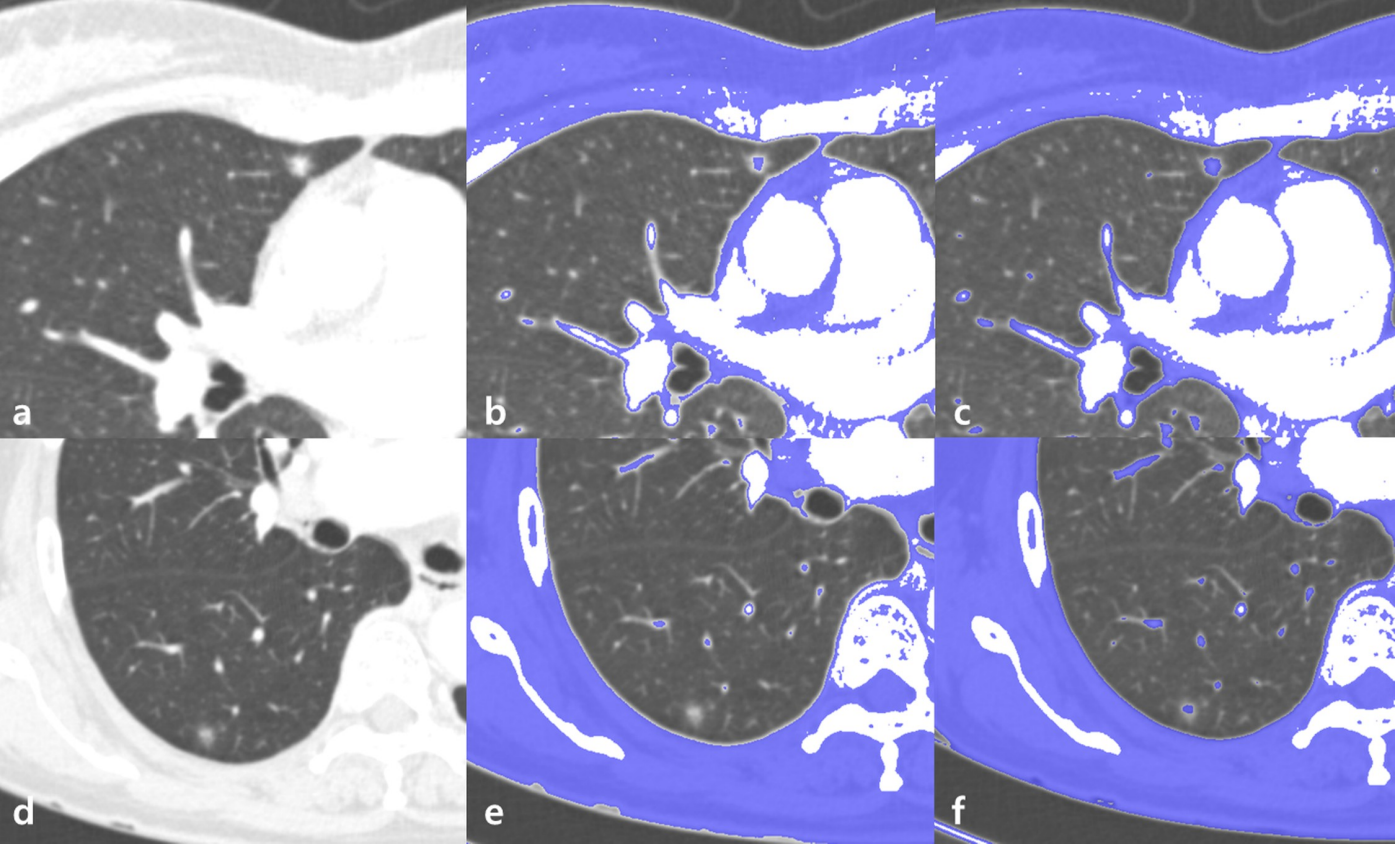
Different subcategorization of ground-glass opacity nodules (GGNs) according to different thresholds: Classifying GGN according to two thresholds. (a) Axial computed tomography (CT) image revealing GGN in the right middle lobe. (b) Axial CT images demonstrating areas above the high threshold (-160 Hounsfield units [HU]) (blue area). The GGN is identified as a part-solid GGN with a solid portion inside. (c) Axial CT images demonstrating areas above the low threshold (-400 HU) (blue area). The GGN is identified as a part-solid GGN with a solid portion inside. (d) Axial CT image demonstrating another GGN in the right lower lobe. (e) Axial CT images demonstrating areas above the high threshold (-160 HU) (blue area). The GGN is identified as a pure GGN without a solid portion inside. (f) Axial CT images demonstrating areas above the low threshold (-400 HU) (blue area). The GGN is identified as a part-solid GGN with a solid portion inside. As a result, the GGN in (a) is a part-solid GGN in both thresholds, whereas the GGN in (d) is a pure GGN in high threshold and part solid GGN in low threshold.

The solid component of GGNs was defined using two different attenuation thresholds: an area that demonstrated attenuation > -160 HU (high attenuation threshold); and an area that demonstrated attenuation > -400 HU (low attenuation threshold). After overlying the solid portion (above the threshold) using dedicated software, the radiologists manually outlined and segmented the solid portion. According to the presence or absence of a solid portion, GGNs were categorized into pure GGNs (no solid component) and part-solid GGNs (with solid component[s]) for each of the two thresholds. Solid components were considered to be indicators of invasive foci, suggesting MIA or IA (Fig 1).

### Statistical analysis

Continuous variables were summarized as mean ± SD, and categorical variables were summarized as frequencies or percentages. Data distributions were tested using the Shapiro-Wilk testThe sensitivity, specificity, positive predictive value (PPV), negative predictive value (NPV), and accuracy of the high and low attenuation threshold were calculated for the detection of invasive foci based on the assumption that the solid portion on CT images indicated invasive foci on pathology. Interobserver agreement regarding the presence or absence of solid portion was evaluated using Cohen kappa test. A kappa value of 0.00–0.20 indicated none to slight agreement; 0.21–0.40, fair agreement; 0.41–0.60, moderate agreement; 0.61–0.80, good agreement; and 0.81–1.00, excellent agreement. All statistical analyses were performed using commercially available software (SPSS version 20, IBM Corporation, Armonk, NY, USA) for Windows (Microsoft Corporation, Redmond, WA, USA) or SAS version 9.4 (SAS Institute Inc., Cary, NC, USA).

## Results

A total of 86 GGNs in 86 patients (27 male, 59 female; mean age 59.3 ± 11.0 years) were evaluated in this study. The mean size of the tumors was 15.0 ± 7.5 mm. Detailed demographic patient data are summarized in Table 1.

**Table 1:** Demographic data of the 86 patients in this study.

Variables	AIS	MIA	IA
**Number (%)**	10 (11.6%)	19 (22.1%)	57 (66.3%)
**Sex (male: female)**	1:9	6:13	20:37
**Age (years)**	58.1 ± 9.6	57.0 ± 12.5	60.4 ± 10.8
**Diameter (mm)**	8.9 ± 3.8	10.1 ± 4.3	17.6 ± 7.4
**Mean attenuation (HU)**	-611.9 ± 98.7	-508.4 ± 76.2	-404.8 ± 169.2

Data presented as mean ± SD unless otherwise indicated. AIS, adenocarcinoma in situ; MIA, minimally invasive adenocarcinoma; IA, invasive adenocarcinoma; HU Hounsfield units

Among the 86 GGNs, there were 57 cases of IA, 19 of MIA, and 10 of AIS, which were pathologically confirmed in surgical specimens. Using the high attenuation threshold (-160 HU), 44 were categorized as pure GGNs (21 IA, 13 MIA, and 10 AIS), and 42 were categorized as part-solid GGNs (36 IA, 6 MIA and no AIS). With the low attenuation threshold (-400 HU), 13 were categorized as pure GGNs (3 IA, 2 MIA, and 8 AIS), and 73 as part-solid GGNs (54 IA, 17 MIA, and 2 AIS) (Table 2). The kappa values for the solid portion were 0.904 when using high attenuation threshold and 0.837 when using low attenuation threshold.

**Table 2:** Sub-categorization of ground-glass opacity nodules (GGNs) according to high and low thresholds.

High attenuation threshold			Low attenuation threshold		
**Pure GGN**	**AIS**	10 (22.7%)	**Pure GGN**	**AIS**	8 (61.5%)
	**MIA**	13 (29.5%)		**MIA**	2 (15.4%)
	**IA**	21 (47.8%)		**IA**	3 (23.1%)
	**Subtotal**	**44**		**Subtotal**	**13**
**PS-GGN**	**AIS**	0 (0%)	**PS-GGN**	**AIS**	2 (2.7%)
	**MIA**	6 (14.3%)		**MIA**	17 (23.3%)
	**IA**	36 (85.7%)		**IA**	54 (74.0%)
	**Subtotal**	**42**		**Subtotal**	**73**

Data presented as n (%) unless otherwise indicated. AIS, adenocarcinoma in situ; MIA, minimally invasive adenocarcinoma; IA, invasive adenocarcinoma; PS, partly solid

The sensitivity, specificity, PPV, NPV, and diagnostic accuracy for the invasive foci were 55.2%, 100%, 100%, 22.7%, and 60.4%, respectively, using the high attenuation threshold, and 93.4%, 80%, 97.2%, 61.5%, and 91.8%, respectively, with the low attenuation threshold (Tables 3 and 4) [S1 File].

**Table 3:** Diagnostic accuracy of the high threshold for the detection of invasive foci on ground-glass opacity nodules (GGNs).

	IA + MIA	AIS	Total
**Pure GGN**	34	10	44
**Part solid GGN**	42	0	42
**Total**	76	10	86

Data presented as n. AIS, adenocarcinoma in situ; MIA, minimally invasive adenocarcinoma; IA, invasive adenocarcinoma

**Table 4:** Diagnostic accuracy of the low threshold for the detection of invasive foci on ground-glass opacity nodules (GGNs).

	IA + MIA	AIS	Total
**Pure GGN**	5	8	13
**Part-solid GGN**	71	2	73
**Total**	76	10	86

Data presented as n. AIS, adenocarcinoma in situ; MIA, minimally invasive adenocarcinoma; IA, invasive adenocarcinoma

## Discussion

This study demonstrated that the low attenuation threshold (-400 HU) was better than the high attenuation threshold (-160 HU) in determining the solid component of GGNs, which indicates an invasive focus. GGNs can be divided into part-solid and pure according to morphology on CT imaging. Although the solid portions of GGNs have a tendency to represent invasive foci, they do not directly correspond with invasive foci [15]. Visual assessment with qualitative analysis for differential diagnosis of GGNs has well-known limitations, including inter-/intra-observer variations [11,16,17]. To overcome the drawbacks of visual assessments, various types of quantitative analysis of GGNs have recently been reported. The sizes of GGNs correlate with the invasion foci and pathologic results [18]. Lim et al. [19] reported that a difference in mean attenuation value could be observed between invasive and non-invasive adenocarcinomas. Lee et al. [20] suggested -472 HU as the cut-off of mean attenuation value in the evaluation of tumor invasiveness. However, with these methods, it is difficult to define the invasive focus of a GGN directly, although identifying the invasive focus is the easiest way to differentiate AIS from MIA and IA. Furthermore, in the 8^th^ TNM guidelines the T staging of lung cancer depends on the size of the solid portion [21]. For these reasons, direct measurement of the solid portion of a GGN, which indicates an invasive focus, is crucial, and the absolute attenuation threshold to define invasive foci has been applied because CT attenuation is related to the density of the tissue [22]. Matsuguma et al. [12] attempted to define the solid portion of a GGN using a threshold of -160 HU. Ko et al. [13] set the threshold at -188 HU and regarded the part of the nodule that exhibited higher values as the solid component. Recently, Cohen et al [14] used -350 HU, as the threshold when measuring GGNs, because the invasive foci and solid component of the GGN demonstrated the highest agreement for that threshold. Therefore, the optimal threshold for identifying the solid portion of GGNs has not been established. In this study, we attempted to obtain better thresholds for defining the solid portion of GGNs. We used -400 HU as a representative low threshold and -160 HU as a representative high threshold. The high threshold categorized all AIS cases as pure GGNs, but 44.8% of MIA and IA cases were also categorized as pure GGNs. In contrast, with the low threshold, 93.4% of MIA and IA cases were categorized as part-solid GGNs. These findings suggest that with the use of a high threshold, invasive foci may be missed, and a low threshold might be better at distinguishing MIA and IA, which are observed as part-solid GGNs, from AIS, which are observed as pure GGNs.

With the development of CT technology and analysis software, advanced analyses are possible for differential diagnosis of GGNs. Recently, quantitative CT texture analysis has provided diverse potential applications for GGN differentials. Ikeda et al. [23] reported that IA exhibited two peaks on a CT histogram. Son et al. [24] demonstrated that the 75th percentile CT attenuation value could be used to distinguish IA from pre-invasive lesions. Kurtosis, skewness, or entropy of GGNs could be used for differentiating pre-invasive lesions and IA [24–26]. In addition, advanced radiomic features including shape and morphology metrics, Renyi dimensions, geometrical measures, and the gray-level co-occurrence matrix or the gray-level run length matrix might be useful for differential diagnosis of GGNs [27]. However, these complex methods are difficult to apply in daily practice, and further validation is needed [26].

Our study had some limitations, including its single-center, retrospective design and the small sample size. Second, vessels on CT imaging were not perfectly removed due to the limitations of manual image processing. Third, the slice thickness of CT images ranged from 1 mm to 2.5 mm, which may have led to a volume averaging effect in thicker sections of the CT scans [28,29]. Fourth, in most cases, one dedicated pathologist with experience in lung cancer reported the pathologic results, but in some cases, different pathologists with different expertise reported. Fifth, this study did not evaluate longitudinal changes of GGNs, although changes in CT characteristics, including density, are helpful for differential diagnosis [30]. In addition, in this study, attenuation thresholds of -160 HU and -400 HU were compared, but these are representative values of low and high thresholds, and since neither is likely to be the optimal threshold, further evaluation for determining the optimal threshold will be necessary.

In conclusion, a low attenuation threshold was better than a high attenuation threshold in determining the solid components of GGNs, which indicate invasive foci. Quantitative analysis using a low threshold for the solid portions of GGNs could be a simple and accurate method to define invasive foci.

## Supporting information

S1 File Attached file includes data of the pathogolic results in surgical specimens and sub-categorizations of ground-glass opacity nodules according to high and low thresholds. (XLSX)

## Abbreviations

CT: computed tomography
GGN: ground-glass opacity nodules
HU: Hounsfield units
IA: invasive adenocarcinoma
MIA: minimally invasive adenocarcinoma
PPV: positive predictive value
NPV: negative predictive value
